# Impaired healing of cornea incision injury in a TRPV1-deficient mouse

**DOI:** 10.1007/s00441-018-2878-y

**Published:** 2018-07-04

**Authors:** Yuka Nidegawa-Saitoh, Takayoshi Sumioka, Yuka Okada, Peter S. Reinach, Kathleen C. Flanders, Chia-Yang Liu, Osamu Yamanaka, Winston Whei-Yang Kao, Shizuya Saika

**Affiliations:** 1 Department of Ophthalmology, Wakayama Medical University, 811-1 Kimiidera, Wakayama, 641-0012 Japan; 2 School of Ophthalmology and Optometry, Wenzhou Medical University, Zhejiang, China; 3 Laboratory of Cancer Biology and Genetics, Center for Cancer Research, National Cancer Institute, National Institutes of Health, 37 Convent Dr. MSC 4255, Bldg 37, Bethesda, MD 20892 USA; 4 USA School of Optometry, Indiana University, Bloomington, IN 47405 USA; 5 Department of Ophthalmology, University of Cincinnati School of Medicine, Cincinnati, OH USA

**Keywords:** Cornea, TRPV1, Wound healing, Transforming growth factor β, Myofibroblast, Ultrastructure, Mouse

## Abstract

The present study attempts to elucidate the role of TRPV1 cation channel receptor on primary repair in an incision-wounded mouse cornea in vivo. Previous study revealed that blocking TRPV1 suppressed myofibroblast formation and expression of transforming growth factor β1 (TGFβ1) in cultured keratocytes or ocular fibroblasts. Male C57BL/6 (wild-type; WT) mice and male C57BL/6 *Trpv1*-null (KO) mice incurred a full-thickness incision injury (1.8 mm in length, limbus to limbus) in the central cornea of one eye with a surgical blade under general and topical anesthesia. The injury was not sutured. On days 0, 5, and 10, the eyes were enucleated, processed for histology, immunohistochemistry, and real-time RT-PCR gene expression analysis to evaluate the effects of the loss of TRPV1 on primary healing. Electron microscopy observation was also performed to know the effect of the loss of TRPV1 on ultrastructure of keratocytes. The results showed that the loss of *Trpv1* gene delayed closure of corneal stromal incision with hindered myofibroblast transdifferentiation along with declines in the expression of collagen Ia1 and TGFβ1. Inflammatory cell infiltration was not affected by the loss of TRPV1. Ultrastructurally endoplasmic reticulum of TRPV1-null keratocytes was more extensively dilated as compared with WT keratocytes, suggesting an impairment of protein secretion by TRPV1-gene knockout. These results indicate that injury-related TRPV1 signal is involved in healing of stromal incision injury in a mouse cornea by selectively stimulating TGFβ-induced granulation tissue formation.

## Introduction

The maintenance of corneal transparency is dependent on the stroma remaining avascular non-fibrotic and free of persistent pathogenic inflammation. These properties are essential for preserving a well-organized collagenous framework interspersed with keratocytes embedded in a well-defined extracellular matrix (ECM). If the cornea is injured by an incision, epithelial cells and keratocytes increase their expression of wound healing promoting cytokines that hasten this process (Wilson and Kim 1998; Robb and Kuwabara 1964). Keratocytes adjacent to the wound are activated and assume phenotypic change becoming proliferative fibroblast and then myofibroblasts, which hasten wound closure by inducing contraction and remodeling of the ECM (Brodovsky et al. 2000; Meller et al. 2000; Ishizaki et al. 1993; Saika et al. 1993a, b; Tomasek et al. 2002). Epithelial cells migrate over the newly formed fibrin plugs that are invaded by proliferative granulated tissue in the incision. Various upregulated epithelial growth factors cause changes in cell behavior through complex mechanisms involving pro-fibrogenic and pro-inflammatory responses (Saika et al. 2005a, b). These cytokines include TGFβ1 that is one of the most effective promoters of tissue repair and stromal fibrosis during the healing process (Saika 2006).

Transient receptor potential (TRP) receptors are for the most part non-selective cation cell channels that are activated by multiple external and endogenous cues. They constitute a superfamily composed of 28 genes that are subdivided into 7 subfamilies depending on sequence homology and variable cation permeability (Ramsey et al. 2006; Owsianik et al. 2006; Montell et al. 2002; Pedersen et al. 2005). Among them, TRPV1, the capsaicin receptor, is a TRP archetype isoform in the TRPV subfamily. It is a nociceptor eliciting responses to noxious stimuli including chemical irritants, inflammatory mediators, changes in pH, and moderate heat and hypertonicity (Ciura and Bourque 2006; Steen et al. 1992; García-Hirschfeld et al. 1995). Activation of TRPV1 is followed by increases in Na^+^ and Ca ^2+^ permeability and increases in their influx and activation of linked signal transduction pathways. One group of effects elicited by these transient rises includes release of tachykinin neuropeptides (e.g., substance P, calcitonin gene-related peptide, etc.) from sensory nerves and other cell types, i.e., epithelial and mesenchymal cell types (Caterina et al. 1997; Caterina et al. 2000; Davis et al. 2000). Functional TRPV1 expression constitutively promotes proliferation and migration in human, rabbit, and mouse corneal epithelium in vivo. Some of the cell signaling events associated with these responses include TRPV1 transactivation eliciting epidermal growth factor receptor (EGFR) signaling cascades, which in turn lead to global MAPK and Akt/PI-3K pathway stimulation in SV40-immortalized human corneal epithelial cells (Pan et al. 2011). Moreover, TRPV1 signal supports transforming growth factor β1 (TGFβ1)-mediated myofibroblast transdifferentiation of stromal keratocytes and kidney mesenchymal cells, that accounts for corneal opacification and renal fibrosis (Okada et al. 2011; Wang and Wang 2011).

Even though TRPV1 activation promotes corneal tissue repair following an incision injury, the wound healing response induced by a more severe injury corneal alkali burns results instead in persisting inflammation and tissue fibrosis in mice. Its involvement in this unfavorable outcome was confirmed by the observation that the ablation of *Trpv1* gene markedly reduced the severe sights compromising responses caused by alkali burn (Okada et al. 2011). Reciprocal bone marrow transplantation between the wild-type (WT) and *Trpv1* gene knockout (KO) mice showed that the phenotype depended on the chemotactic activity of the stromal resident cells of each of these two genotypes rather than TRPV1 expression on the infiltrating inflammatory cells (Okada et al. 2011). These markedly different TRPV1-induced wound healing outcomes depended on whether the inflammatory response was self-limiting or became chronic. It was suggested that the duration and magnitude of macrophage infiltration was one of the factors determining inflammatory response severity. To address this question, we report here on the role of TRPV1 activation in wound healing process in a less-inflammatory simple incision corneal wound healing model in mice. Because we previously reported that phenotype of healing in alkali-burned cornea depends on keratocyte genotype rather than inflammatory cells, we here investigated the ultrastructure of a KO keratocyte as well as examined cellular phenotypes by using immunohistochemistry and gene expression analysis.

## Materials and methods

Experiments in vivo were approved by the DNA Recombination Experiment Committee and the Animal Care and Use Committee of Wakayama Medical University, and conducted in accordance with the Association for Research in Vision and Ophthalmology Statement for the Use of Animals in Ophthalmic and Vision Research.

### Incision injury in the central cornea

Adult male C57BL/6 (WT) mice and adult male KO mice of C57BL/6 background (RIKEN, Tokyo, Japan) (Okada et al. 2011) were systemically and topically anesthetized as previously reported (Saika et al. 2005a, b). A full-thickness incision (1.8 mm in length, limbus to limbus, temporal–nasal) was produced in the central cornea of one eye with a surgical blade (Satin Crescent, Alcon, Fort Worth, TX) following administration of a mydriatic. Subsequently, ofloxacin ointment was topically administered immediately after inducing an ocular injury, which was allowed to heal without being sutured for up to 10 days. On days 5 and 10, eyes were enucleated and fixed in 4% paraformaldehyde for 48 h. Twenty-six or 32 WT and 26 or 34 KO eyes were prepared for histology on days either 5 or 10, respectively. Fixed specimens were embedded in paraffin and then deparaffinized sections (7 μm thickness) were stained with hematoxylin and eosin (HE) and immunohistochemistry and observed under light microscopy as previously reported (Saika et al. 2005a, b). The number of eyes whose wound had closed was identified based on histological results and statistically analyzed as described below.

### Ultrastructural observation

Ultrastructural observation was also performed to analyze the effect of TRPV1 gene knockout on ultrastructure of the corneal cells. WT and KO uninjured corneas and those at day 5 post-incision were routinely processed for routine transmission electron microscopy as previously reported (Saika et al. 1993a, b).

### Immunohistochemistry

Deparaffinized sections were processed for immunohistochemistry as previously reported (Saika et al. 2005a, b). Antibodies used are listed in Table 1. Antibody-specific tissue antigen interactions were identified based on peroxidase-conjugated secondary antibody reactions visualized with 3,3′-diaminobenzidine (Nichirei Biosciences, Tokyo, Japan) and nuclei counterstaining with methylgreen. Secreted active form of TGFβ1 was immunostained as previously reported with a specific antibody produced by us (Flanders et al. 1991; Flanders et al. 1989). Specimens were observed under light microscopy (BX50, Olympus, Tokyo, Japan).

**Table 1:** Antibodies used

Rabbit polyclonal anti-TRPV1 antibody (1:500; Neuromics)	
Mouse monoclonal anti-α-SMA antibody (1:200; Neomarker, Fremont, CA)	
Rabbit polyclonal anti-SM22 alpha antibody (1:200; Abcam, Cambridge, UK)	
Rabbit polyclonal MPO antibody (1:100; Thermo scientific, Fremont, CA)	
Rat monoclonal anti-macrophage F4/80 antibody (1:50; BMA Biomedicals, August, Switzerland)	
Active form of TGFβ1 antibody (Flanders et al. 1989)	

*TRPV1* transient receptor potential vanilloid subtype 1, *αSMA* α-smooth muscle actin, *MPO* myeloperoxidase

### Real-time reverse transcription-polymerase chain reaction analysis of gene expression level in vivo

Healing incision-injured WT and KO mice corneas on day 3 were processed for total RNA extraction as previously reported (Saika et al. 2005a, b). RNA samples were obtained from 80 corneas of each genotype on days 0 and 3. Each sample contained four pooled corneas at each time point. Taqman real-time reverse transcription-polymerase chain reaction (RT-PCR) (TaqMan one-step RT-PCR master mix reagents kit, Applied Biosystems, Foster City, CA) was performed to assay the mRNA expression level of collagen Ia1 and α-smooth muscle actin (αSMA) by using Applied Biosystems 7300 Fast real-time RT-PCR system (Applied Biosystems, Foster City, CA) as previously reported (Saika et al. 1993a, b; Tomasek et al. 2002).

### Statistical analysis

In HE histology, the number of eyes with and without closure or just covered with newly formed granulation tissue was analyzed by using the Fisher’s exact test with significance as *p* < 0.05. Data obtained from real-time RT-PCR analysis were shown as means plus/minus standard deviation (S. E.). ANOVA was employed in multiple group comparisons. The value of *p* < 0.05 was considered as statistically significant (Table 2).

**Table 2:** Primers and oligonucleotide probes used

Primer	Oligonucleotide probe
α-SMA	Mm01204962_gh
F4/80	Mm00802524_ml
Collagen 1a1	Mm00801666_gl
GAPDH	Mm03302249_gl

*αSMA* α-smooth muscle actin; GAPDH, glyceraldehyde-3-phosphate dehydrogenase

## Results

### Healing of KO and WT mouse eyes following an incision injury in cornea

**Fig. 1:**
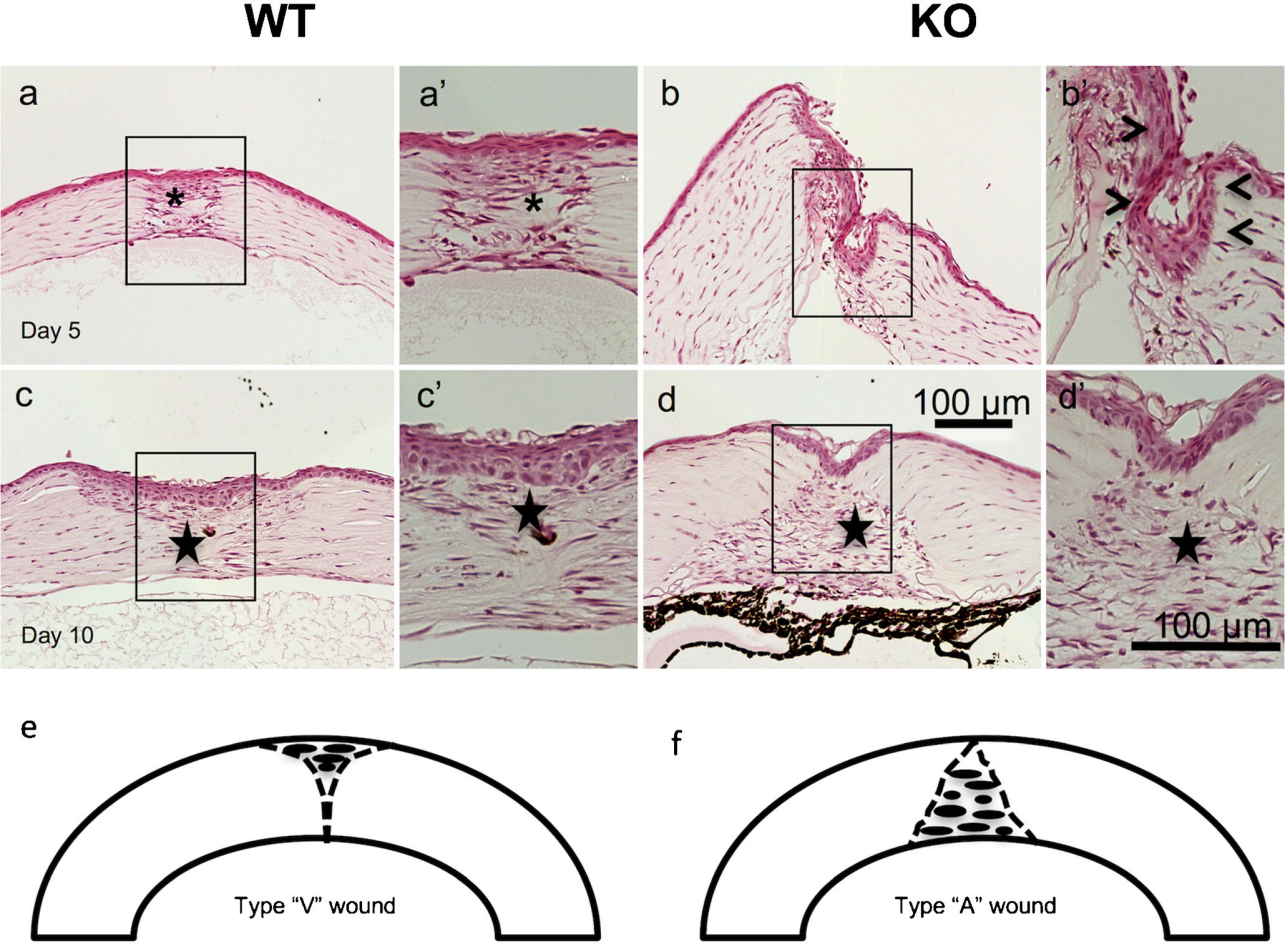
Hematoxylin and eosin staining of a healing incision-injured wild-type (WT) or a TRPV1-null (KO) mouse cornea. At day 5 post-injury, the incision injury was sealed with cellular component-rich granulation tissue (asterisk) in a WT cornea (**a**), while in a KO specimen the stroma of the incision wound does not adhere to each other (**b**). Frames (a’ and b’) indicate the boxed areas of frames (**a** and **b**). In a WT granulation tissue (asterisk) in the wound is occupied with elongated cells and accumulation of matrix (a’). In a KO cornea, the epithelium (arrowheads) is grown down on the cut surface of the stromal wound (b’). At day 10, the wound is healed with scar tissue (star) in both WT and KO corneas (**c**, **d**). Frames (c’ and d’) indicate the boxed areas of frames (**c** and **d**), respectively. Cell distribution in the scar tissue seems more intense in a KO tissue as compared with a WT scar. Schemes that explain the shape of the scar tissue at day 10 (**e**, **f**). “Type V wound” indicates that the healing pattern with scar tissue observed beneath the epithelium, i.e., healing pattern shown in frame (***c***) and that with scar tissue greater in the deep stroma above the endothelium as “type A healing” as in the frame (**d**). Bar 100 μm

Lacking *Trpv1* expression was not associated with any detectable histological abnormalities in a mouse eye. As *Trpv1* undergoes upregulation during healing, following either corneal epithelial or stromal injury (Okada et al. 2011), its role of TRPV1 in this process was examined in an incision injury model. At day 5, the stromal injury was sealed (or closed) with a granulated tissue in 23 of 26 WT mice, while the injury remained open in 16 out of 26 KO corneas (Fig. 1). In the KO cornea specimens with patent wounds, the migrating epithelium covered the cut surface of the stroma in KO corneas. Namely, the incidence of injury closure was greater in WT mice as compared with KO mice at day 5 as analyzed by using the Fisher’s exact test (*p* = 0.026). At day 10, the incision was sealed with healing stromal tissue in 31 or 31 of 32 WT or 34 KO mice, respectively. Therefore, loss of TRPV1 gene function did not affect a change in the stromal healing response (Fig. 1).

Although the incidence of wound closed was similar between WT and KO groups at day 10, the newly formed granulation histology tissue differed between the two groups. We designated this better healing pattern having residual scar tissue beneath the epithelium as “type V healing.” On the other type of healing pattern with more scar tissue atop, the endothelium is referred to as “type A healing.” Type V healing (vs. the type A type) was observed in 14 out of 19 WT mice, in contrast 4 out of 23 in KO mice. Therefore, the incision wound of KO mice healed more prevalent with type A scar (Fig. 1(e, f)).

### Ultrastructural observation

**Fig. 2:**
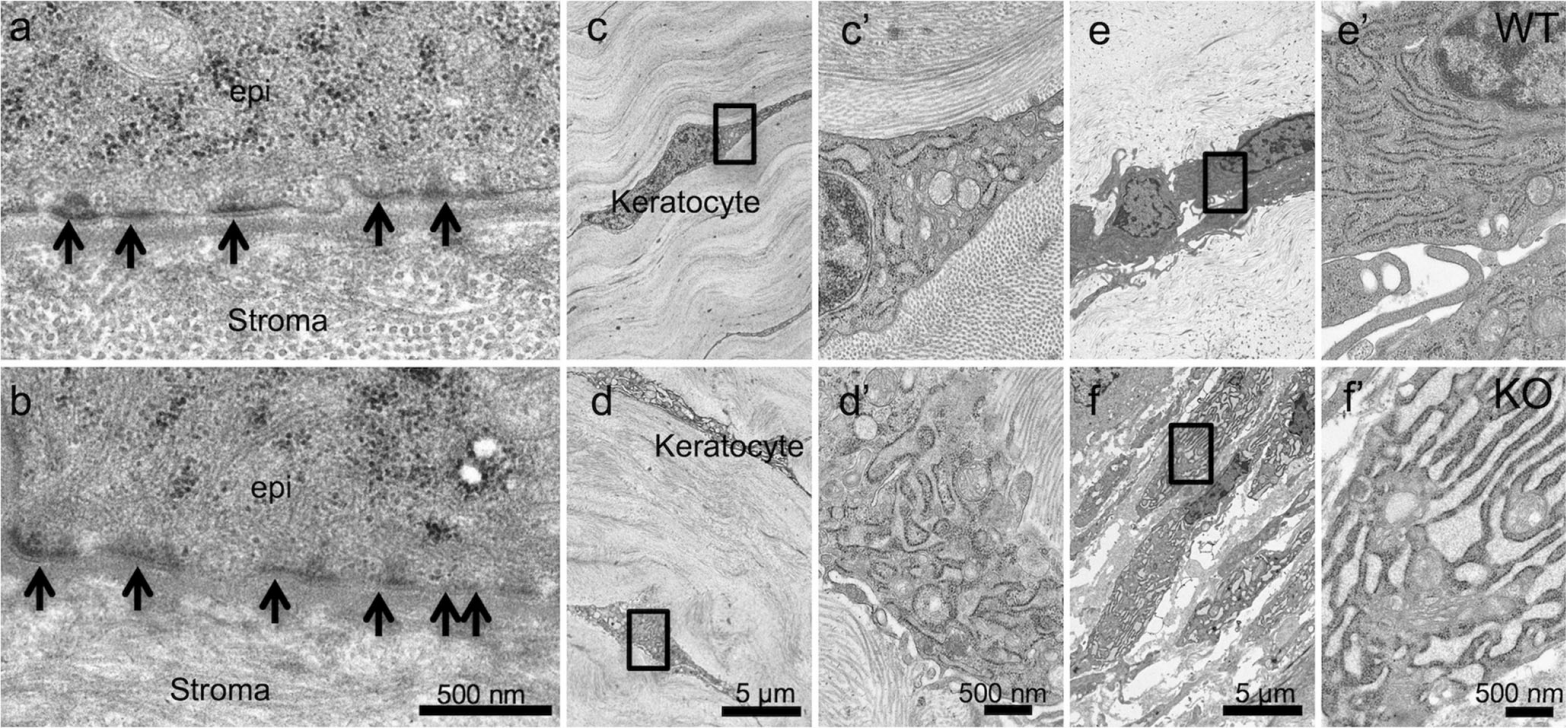
Ultrastructure of an uninjured cornea and that during healing at day 5 post-incision injury of either wild-type (WT) or a TRPV1-null (KO) mouse. The basement membrane with well-formed desmosomes (arrows) is observed in either WT (**a**) or KO (**b**) mouse without obvious difference. Stroma is composed of collagen lamellae and keratocytes among the collagen fibers in both WT (**c**) and KO (**d**) corneas. Frames (**c**’ and d’) show higher magnification pictures of the quadrilateral areas in Frames (**c** and **d**), respectively. Endoplasmic reticulum (ER) seems more developed in the cytoplasm of a KO keratocytes (**d**’) as compared with a WT cells (**c**’). Frames (**e**’ or **f**’) indicates higher magnification picture of the quadrilateral areas in frames (**e** or **f**), respectively. ER seems more developed and more dilated in the cytoplasm of a KO keratocytes (**f**’) as compared with a WT cells (**e**’)

Stroma and epithelial basement membrane in an uninjured cornea and that 5 days after incision were observed under transmission electron microscopy. Structure of cell-cell junction in the epithelium was not affected by the loss of TRPV1 (data not shown). Stromal lamellae composed of collagen fibers covered by non-keratinizing stratified the epithelium with well-developed basement membrane with desmosomes in both WT and KO corneas were observed (Fig. 2(a, b)). Keratocytes were observed in the stroma (Fig. 2c–f). High magnification observation showed a typical fibroblastic intracellular organelle, i.e., endoplasmic reticulum, mitochondria, and nucleus in a WT uninjured tissue (Fig. 2(c)). Different from a WT keratocyte (Fig. 2(c’)) almost all the cells in an uninjured KO stroma had an extensively dilated endoplasmic reticulum in the cytoplasm (Fig. 2(d, d’)). Such finding in the endoplasmic reticulum was more prominent in a KO healing keratocytes as compared with a WT healing cells at day 5 post-wounding (Fig. 2(e, e’, f, f’)).

### Appearance and distribution of myofibroblasts in tissues

**Fig. 3:**
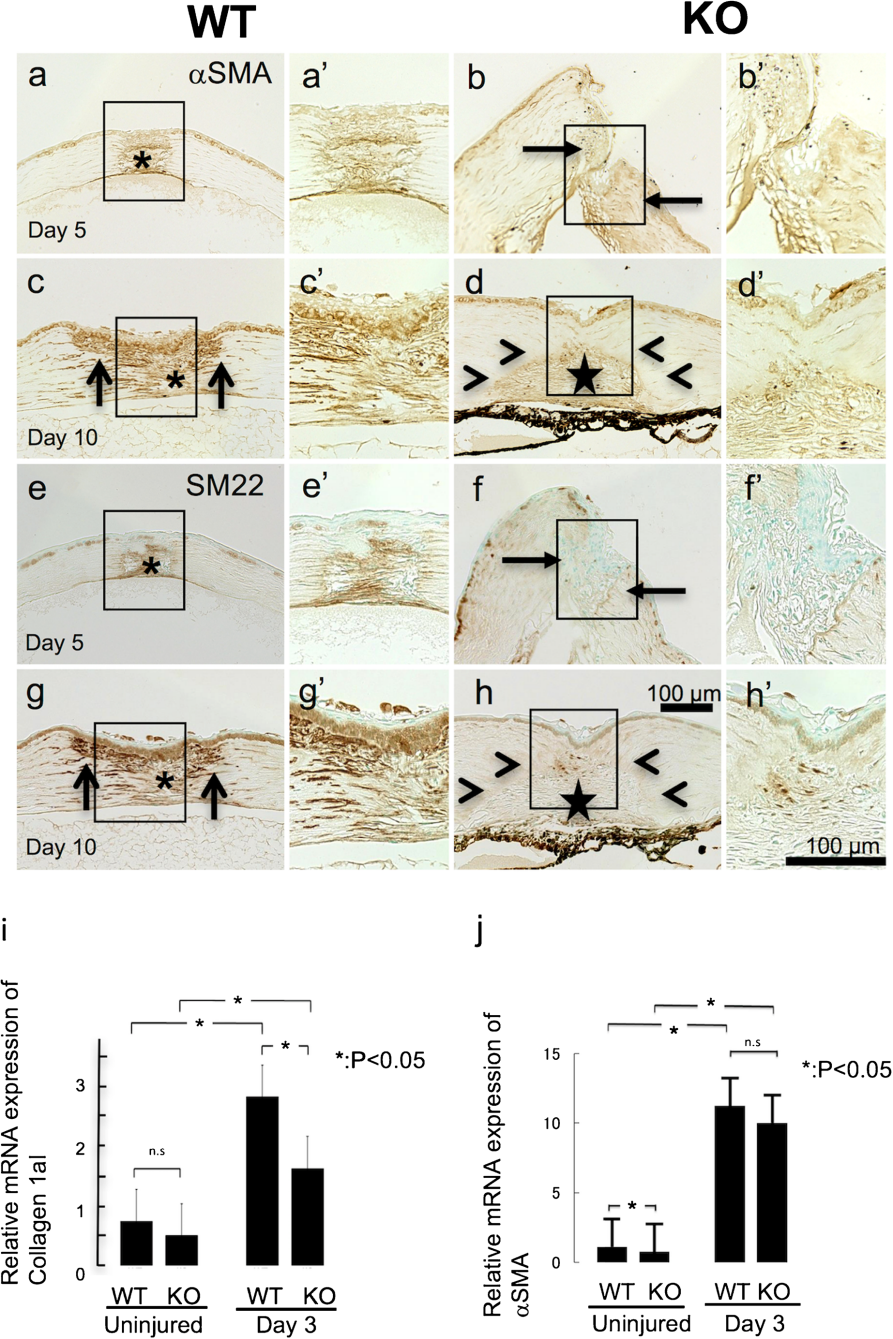
Detection of myofibroblasts in a healing incision-injured cornea of a wild-type (WT) or a TRPV1-null (KO) mouse based on gene expression analysis on collagen 1a1 and α-smooth muscle actin (αSMA) and immunohistochemistry for αSMA and SM22 in healing corneas At day 5, the healing granulation tissue formed in the wound is occupied with αSMA-labeled myofibroblasts (asterisk) in a WT cornea (**a**). On the other hand, few myofibroblast is observed in a KO stroma, even in the area adjacent to the incision (arrows) (**b**). At day 10, myofibroblasts are distributed not only in the scar tissue (asterisk) but also in the stroma adjacent to the incision injury (arrows) in a WT tissue (**c**). Myofibroblasts are also seen in the granulation tissue (star) formed in the incision in a KO stroma, but the immunoreactivity seems less intense in a KO tissue as compared with a WT cornea (**d**). It is to be noted that the stroma adjacent to the granulation tissue is free from myofibroblasts (arrowheads) in a KO corneal stroma (**d**). Panels (**e**–**h**) show the expression pattern of SM22, another marker for myofibroblast. SM22 expression pattern is similar to that for αSMA indicated in panels (**a**–**d**). mRNA expression of collagen 1a1 is upregulated by the injury at day 3 as compared with baseline expression level (**i**). Such upregulation is counteracted by the loss of TRPV1. mRNA expression of αSMA is suppressed in an uninjured cornea by TRPV1 gene ablation. However, upregulation of αSMA is not affected by TRPV1 knockout at day 3 (**j**)

Type I collagen is one of the major extracellular matrix components of the stromal collagen fibrils and it is also the major constituent of the fibrotic tissue caused by injury and pathogenesis. We therefore first determined if lacking *Trpv1* gene expression alters *Col1a1* mRNA expression in a healing cornea. The *Col1a1* mRNA expression level was invariant between naive uninjured cornea of WT and KO mice. Three days after an incision, *Col1a1* mRNA gene increased in injured corneas of both groups and it reached a higher level in WT than KO mice (Fig. 3(i)).

On the other hand, real-time RT-PCR showed that basic (uninjured) and increased expression levels of αSMA mRNA (injured) were unaffected by the loss of TRPV1 in KO mice in comparison to that of injured corneas of WT mice healed for 3 days, respectively (Fig. 3(j)). Under healing process in a more severely injured stroma with a cross-shaped double incision, upregulated expression of αSMA was subdued by the loss of TRPV1 (data not shown).

Immunohistochemical examination showed a clear difference of the distribution pattern of myofibroblasts in healing tissues between WT and KO mice. Myofibroblasts labeled for αSMA appeared at day 5 post-incision in a WT cornea (Fig. 3(a)). Myofibroblasts were observed in the granulation tissue as well as in the stroma adjacent to the incision wound edge. On the other hand, αSMA-labeled cells were quite fewer in the stroma adjacent to the wound of a KO mouse at this time point (Fig. 3(b)). On day 10, a large number of αSMA-labeled myofibroblasts appeared in the newly formed granulation tissue and stroma adjacent to the incision of WT mice (Fig. 3(c)). Expression of αSMA seemed to increase at lesser degree in the granulation tissue of KO mice at day 10 as compared to that of WT mice (compare Fig. 3(d, c)) 10 days post injury; unlike WT, stroma adjacent to the granulation tissue was free of myofibrobalsts (Fig. 3(d)). Expression pattern of smooth muscle (SM)-specific SM22 protein (SM22) also showed a similar expression pattern as αSMA (Fig. 3(e, h)).

### Infiltration of inflammatory cells

**Fig. 4:**
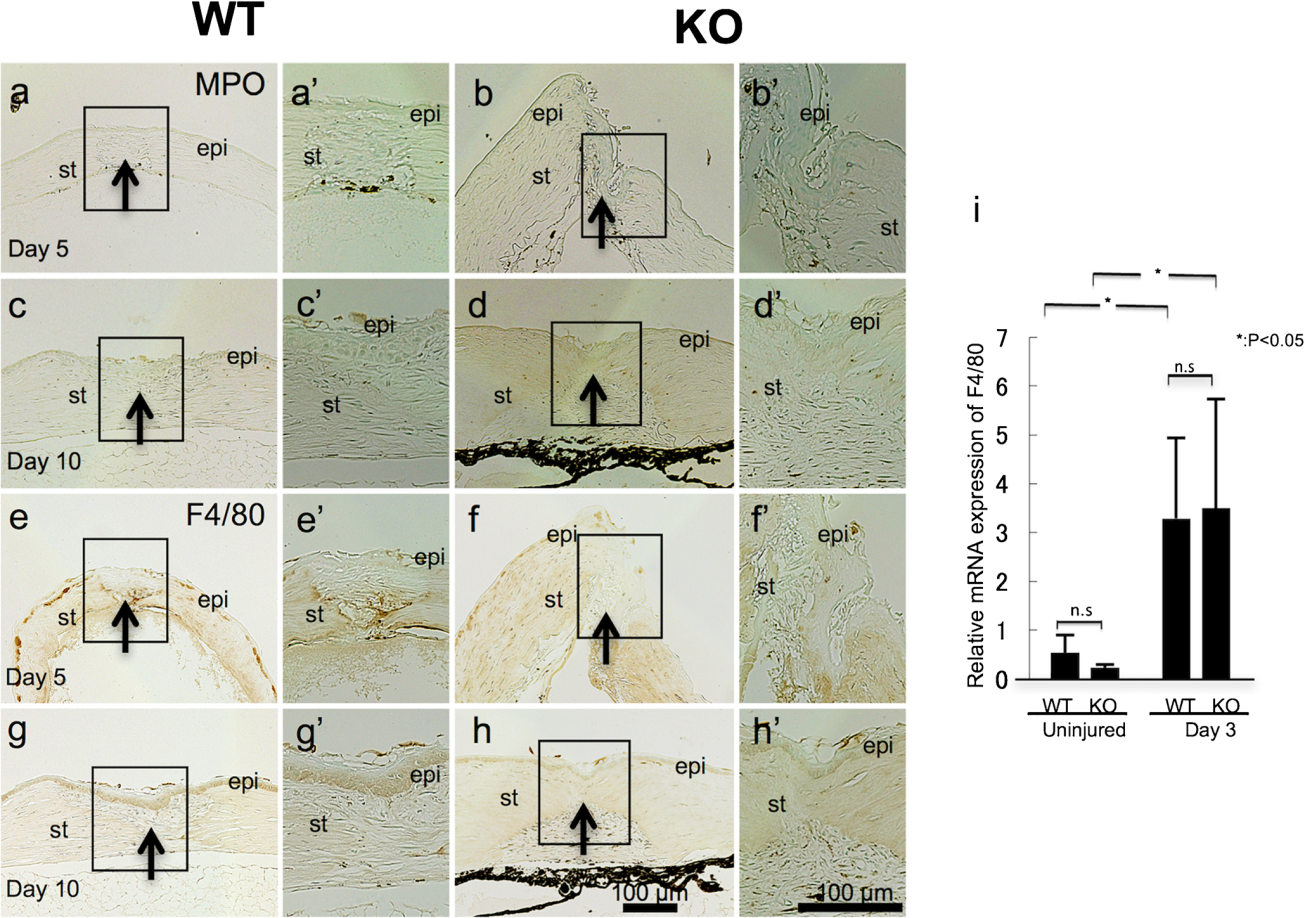
Immunohistochemical detection of neutrophils (myeloperoxidase-positive) and macrophages (F4/80-labeled) in a healing wild-type (WT) or a TRPV1-null (KO) mouse incision-injured cornea. Infiltration of myeloperoxidase-expressing neutrophils was not obviously observed in injured healing tissue of both WT (**a**, **c**) and KO (b, d) mice at day 5 (**a**, **b**) and 10 (**c**, **d**). Macrophage invasion was also evaluated by immunohistochemistry for F4/80 antigen. At day 5, abundant F4/80-labeled macrophages were detected in the granulation tissue formed in the wound of a WT mouse (**e**), while very few immune-labeled cells were seen in a KO cornea (**f**). At day 10, macrophage invasion was much reduced in the stroma of both genotypes of mice (**g**, **h**). Frames (a’–h’) indicate the boxed areas of each frames that indicate the higher magnification pictures of the immune-labeled cells in the stroma. *epi* epithelium, *st* stroma, *arrows* at the site of the original injury. Bar 100 μm. Real-time RT-PCR does not show difference of the expression of F4/80 macrophage antigen between two genotypes in an uninjured cornea as well as that at day 3 post-injury (**i**)

Inflammatory cell infiltration during wound healing provides an essential contribution to this process through expressing various cytokines/growth factors promoting this response. Infiltration of myeloperoxidase-expressing neutrophils was not apparent in injured healing tissue of both genotypes at day 5 and 10 (Fig. 4(a–d, a’–d’)). Macrophage invasion was evaluated by monitoring changes in F4/80 antigen expression levels, which were undetectable in WT and KO corneas immediately following injury (data not shown). On the other hand, at day 5, abundant F4/80-labeled macrophages were detected in the granulation tissue formed in the wound of a WT mouse (Fig. 4(e, e’)), while very a few immune-labeled cells were seen in a KO cornea (Fig. 4(f, f’)). At day 10, macrophage invasion was much reduced in both the WT and TRPV1 KO stroma (Fig. 4(g, g’, h, h’)). Real-time RT-PCR showed that expression level of mRNA of F4/80 increased by 3 days after injury in both WT and KO mice, but there was no difference between WT and Trpv1 KO mice, suggesting that absence of TRPV1 did not affect the infiltration of macrophages into the injured corneas (Fig. 4(i)).

### Expression of active form of TGFβ1

**Fig. 5:**
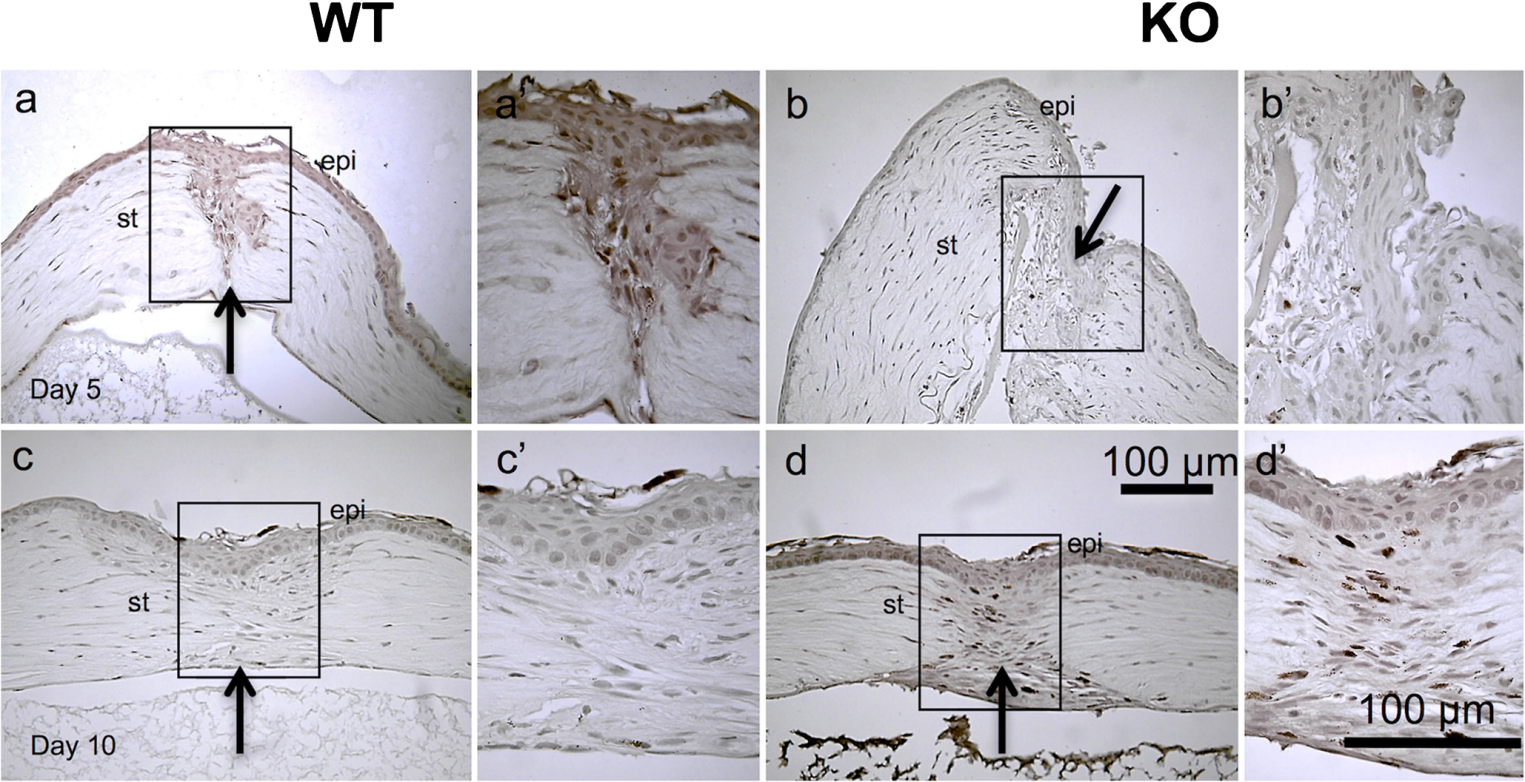
Immunohistochemical detection of active form of transforming growth factor β1 (TGFβ1) expression in a healing wild-type (WT) or a TRPV1-null (KO) mouse incision-injured cornea. Expression of active form of TGFβ1 protein is observed in both epithelium and cells distributed in the stroma (either keratocytes or macrophages) at day 5 post-injury (**a**), while it is much less intense in both epithelium and cells in the stroma by the loss of TRPV1 (**b**). Panels (a’ and b’) are the boxed areas of frames (**a** and **b**), respectively. High magnification observation clearly shows the difference of the staining level for active TGFβ1 in the epithelium between WT and KO mice. At day 10, in turn, its expression turns to regress in a WT tissue (**c**), while it increases to a certain extent in a KO tissue (**d**). High magnification observation shows that TGFβ1 staining in cells in the stroma adjacent to the injury are more marked in a KO cornea (d’) than in a WT tissue (c’). *epi* epithelium, *st* stroma, *arrows* at the site of the original wound. Bar 100 μm

Expression of active form of TGFβ1 in an incision-injured cornea was evaluated by performing immunohistochemistry with a selective anti TGFβ1 antibody (Saika et al. 2005a; Okada et al. 2011; Flanders et al. 1991; Flanders et al. 1989; Thompson et al. 1989). Our previous study showed that this form of TGFβ1 is not present in a healthy mouse cornea (Tomoyose et al. 2015; Saika. 2004). On day 5 after an injury, the TGFβ1 active form was detected in the epithelial and stromal keratocytes or macrophages in WT mice (Fig. 5(a, a’)), but not in that of KO mice (Fig. 5(b, b’)). At day 10, active TGFβ1 expression had regressed in WT mouse cornea (Fig. 5(c, c’)), while the KO corneas had significantly high level of TGFβ1 present in the epithelium and stromal cells (Fig. 5(d, d’)). The observation suggests that ablation of TRPV1 resulted in a delayed activation and/or expression of TGFβ1 induced by injury.

## Discussion

In the present study, we showed that the healing of the full-thickness incision in the cornea was impaired by the loss of *Trpv1* gene function in mice. Myofibroblast formation and accumulation of the extracellular matrix along with activation of *Col1a1* gene expression contribute to the effectiveness of stromal wound healing, tissue fibrosis, and formation of granulation tissue adjacent to the wound. The wound of WT mice was sealed with a granulation tissue containing a number of myofibroblasts at day 5, while the wound closure was significantly delayed in KO mice in which the granulation tissue was not seen at 5 days, but at 10 days of injury. Myofibroblast formation induced by injury occurs as a consequence of injury accompanied by stepwise keratocyte transdifferentiation via transition from quiescent keratocytes, activate proliferative fibroblasts, and terminally differentiated myofibroblasts. The magnitude of this response to injury may be related to the extent of both extracellular matrix remodeling and tissue contraction during tissue repair (Brodovsky et al. 2000; Meller et al. 2000; Ishizaki et al. 1993; Saika et al. 1993a, b; Tomasek et al. 2002). Although KO granulation tissue contained myofibroblasts at day 10, there were few myofibroblasts detected in the stroma adjacent to the incision (outside the granulation tissue). This helps explain why the healing process was impaired in the KO mice. This finding is further supported by the result showing that the loss of *Trpv1* gene caused a reduction of *Col1a1* mRNA expression, the major corneal stromal matrix structural component. By day 5, primary corneal stromal incision injury wound closure occurred in WT mice, but is delayed until day 10 in KO mice.

We then observed ultrastructure of keratocytes because our previous study showed that loss of TRPV1 directly affects keratocyte characteristics, i.e., impaired myofibroblast transformation and less expression of wound healing-related components (Okada et al. 2011). Transmission electron microscopy in the current study showed extensively dilated endoplasmic reticulum in the cytoplasm of KO keratocytes as compared with WT cells both under uninjured or injured conditions. The exact relationship between such ultrastructure of KO keratocytes and attenuation of the wound healing-related behaviors of the cells remain to be clarified. However, our previous study suggested that dilation of endoplasmic reticulum impairment might be caused by an impairment of protein secretion from the endoplasmic reticulum (Saika et al. 1992; Saika. 1992; Sukumaran et al. 2016)

TGFβ1 is an essential mediator of the changes required for inducing myofibroblast transdifferentiation (Garamszegi et al. 2010; Scaffidi et al. 2004; Evans et al. 2003; Margadant and Sonnenberg 2010). We showed in our previous study that inhibition of TRPV1 activity impaired myofibroblast formation in ocular fibroblast culture with TGFβ1 as described above (Okada et al. 2011). Both suppressed susceptibility of the cell against TGFβ1 stimulation and reduced TGFβ1 level in tissue might explain the current findings of the defect in myofibroblast formation. TGFβ-induced myofibroblast formation highly depends on a positive feedback loop where pSmad2-induced ROS activates TRPV1 in corneal fibroblasts in vitro (Yang et al. 2013). In the same report, TRPV1 also activates p38 that further enhances the activation of Smad2, thus it establishes a feed forward loop that augments and sustains the activation of Smad2 signaling cascade for myofibroblast development (Yang et al. 2013). Real-time RT-PCR analysis in the healing, incision-injured, cornea showed no difference of αSMA mRNA expression level between WT and KO tissues at day 3. However, more myofibroblasts, as detected by immunostaining for αSMA and SM22, were observed in the stroma adjacent to the incision wound of WT cornea. The discrepancy of the degrees of in vivo myofibroblast appearance examined by real-time RT-PCR and immunohistochemistry is to be clarified in future studies. A pro-fibrotic effect with activation of Smad2/3 signals of the loss of TRPV1 was reported in a mouse model of deoxycorticosterone acetate-salt-induced renal fibrosis and dysfunction (Wang and Wang 2011). Thus, in vivo αSMA gene expression regulation in a cell could not be similar to that in vitro condition. Rho/ROCK signal, that is essential to the formation of αSMA-positive stress fibers, is reportedly suppressed by the loss of TRPV1. There was, therefore, a possibility that upregulation of αSMA expression might not thoroughly concur along with cytoskeletal fibers of αSMA and SM22 formation in tissue of a KO healing stroma (Li et al. 2015b; Sandbo et al. 2011; Li et al. 2015a).

Although at 10 days post-injury there was no longer a significant difference in the incidence of the cornea with primary wound closure, morphological development of the granulation tissue became dissimilar between the KO and the WT mice. The exact reason why the healing pattern in the stroma differs in the presence or absence of TRPV1 requires future studies for clarification. The heterogeneity of the morphology of the granulation tissue of “type V healing” of WT and “type A healing” of KO cornea could be related to the source of TGFβ1 in issue. Because granulation tissue formation reportedly might be related to the amount of TGFβ in tissue, we expected that the “V-shaped” granulation tissue might depend on the epithelium-derived TGFβ1. On the one hand, “type A healing” in a KO mouse at day 10 could be the outcome of delayed stromal healing with less TGFβ1 expression in the epithelium and resultant fewer myofibroblasts. To test this hypothesis, we conducted immunohistochemistry for active form of TGFβ1 in tissue. TGFβ1 performs its activity after activation by dissociation of its latency-associated components (Saika et al. 2005b; Okada et al. 2011; Flanders et al. 1991; Flanders et al. 1989; Thompson et al. 1989). TGFβ1 active form expression of both corneal epithelial and resident stromal cells was much suppressed in KO cornea as compared with its level in WT tissue. At 5 days of injury in the wounded KO mouse corneas, there is little active TGFβ1 present in the epithelium and stromal cells adjacent to the wound edge is consistent with our previous study in that lacking TRPV1 suppresses expression of TGFβ1 in cultured ocular fibroblasts (Okada et al. 2011). Our unpublished data showed that blocking TRPV1 by a chemical antagonist suppressed mRNA expression of TGFβ1 in an SV40 immortalized corneal epithelial cells, although the cell line was not identical to the primary cultured cells. At day 10, delayed upregulation of active TGFβ1 seen at 10 days of incision could account for the defect of epithelial closure with a delayed formation of granulation tissue at 5 days of healing. This may be in part explained why “type A healing” pattern is seen in KO mice.

Infiltration of neutrophils and macrophages into the wound site did not significantly contribute to the KO phenotype because there was no significant accumulation of these two cell types in both WT and KO tissues at any time point examined. Our previous study showed that macrophage infiltration was suppressed by lacking TRPV1 in the healing of alkali-burned cornea. These findings suggest that *Trpv1* gene ablation could reduce macrophage invasion in a severely injured cornea, but not in a cornea with a single incision.

Taken together, the loss of *Trpv1* gene function retarded primary closure of an injured cornea with a single incision. Delayed upregulation of active form of TGFβ1 in KO epithelium and stroma could be related to the reason of the heterogeneity of the shape of the granulation tissue formed in the incision injury. To regulate TRPV1 activity could be a strategy to modulate the process of stromal healing following injuries or surgeries for the purpose of regulation of scarring and disturbance of the corneal shape.
